# Prognostic comparison between radical prostatectomy and radiotherapy in prostate cancer patients at different stages and ages

**DOI:** 10.18632/aging.203198

**Published:** 2021-06-29

**Authors:** Fei Wang, Yuanming Fan, Xiaojian Yin, Lian-Wen Qi, Gaoxiang Ma, Qinbo Yuan

**Affiliations:** 1 Clinical Metabolomics Center, China Pharmaceutical University, School of Traditional Chinese Pharmacy, Nanjing, China; 2 Department of Urology, The Fifth People’s Hospital of Wuxi, Wuxi, China

**Keywords:** prostatic neoplasms, radiotherapy, ages of onset

## Abstract

Radical prostatectomy (RP) and radiotherapy (RT) are both evidence-based nonconservative treatments for prostate cancer (PCa). However, which treatment is better remains controversial. This study aimed to compare the prognostic difference between radical prostatectomy (RP) and radiotherapy (RT) in PCa patients at different stages and ages. Two independent PCa cohorts (the Surveillance, Epidemiology, and End Results, SEER; and the Prostate, Lung, Colorectal, and Ovarian, PLCO) were employed. Cox regression was used to calculate the hazard ratios (HRs) and the corresponding 95% confidence intervals (CIs). In both cohorts, patients who received RT exhibited a worse prognostic outcome than those who underwent RP. When stratified analysis was performed by tumor node metastasis (TNM) stage and age at diagnosis in the SEER cohort, the HR of RT versus RP for overall survival increased with TNM stage but decreased with age. Specifically, PCa patients in stage I in the age range of 55–84 years, stage IIA at 70–85+ years, and stage IIB at 75–85+ years had better survival with RT than RP patients (*p* < 0.05). In contrast, patients in stages IIA, IIB, III and IV with respective age ranges of 55–64 years; 50–74 years; 55–59, 65–74 years; and 45–74 years showed worse survival with RT compared with RP (*p* < 0.05). These findings were partially validated in the PLCO dataset. Our results indicated that the choice between RT and RP should be guided by TNM stage and age. These findings may facilitate counseling regarding the prognostic effect of RT and RP for PCa patients.

## INTRODUCTION

Prostate cancer (PCa) is the second most common cancer and the most common cause of cancer death among American men [1]. According to cancer statistics in 2018, the incidence of PCa is the fifth most common cancer in China [2]. Radical prostatectomy (RP) and radiotherapy (RT) are both evidence-based nonconservative treatments for PCa [3, 4]. Despite many publications comparing the prognosis between these two treatments, the better option remains unclear for PCa patients [5–9]. The prognosis of PCa patients is correlated with the age at diagnosis [10]. Our previous study also observed that the age at diagnosis played a key role in the prognosis of PCa patients who received RT [11]. In addition, the tumor node metastasis (TNM) stage exerts much influence on PCa prognosis. However, previous studies placed little emphasis on age and TNM stage, which possibly leads to bias.

This study aimed to compare the prognostic difference between RP and RT in PCa patients at different ages and various stages of this disease. Two independent PCa cohorts were employed, namely, the Surveillance, Epidemiology, and End Results (SEER) and the Prostate, Lung, Colorectal, and Ovarian (PLCO) cohorts. The results from this work would benefit clinicians and PCa patients in choosing between RT and RP.

## METHODS

### Study population

For the SEER cohort, a case listing session was obtained from the SEER program using SEER*Stat 8.2.1 (http://seer.cancer.gov/) [12]. The current SEER project includes 17 population-based cancer registries that represent approximately 28% of the US population. SEER data are available to the public for the purpose of studying cancer-based epidemiology. The SEER cohort was approved by the Surveillance Research Program in NCI's Division of Cancer Control and Population Sciences, with the application number 10809-Nov2017.

The Prostate, Lung, Colorectal, and Ovarian (PLCO) Cancer Screening Trial is a randomized trial. This study aimed to evaluate the impact of screening modalities on cancer mortality [13]. Briefly, 154,952 individuals aged 55–74 years were recruited via 10 centers from the US between 1993 and 2001. All participants provided written informed consent, and the study was approved by the Institutional Review Boards at the National Cancer Institute and the 10 recruitment centers. We applied the PLCO cohort to the National Cancer Institute Cancer Data Access System. The project ID is PLCO-411.

Participants provided written informed consent as stated by the SEER and PLCO cohorts. The China Pharmaceutical University Ethics Committee approved the study. This study complied with the Declaration of Helsinki and the regulations of the China Pharmaceutical University Ethics Committee (http://kjc.cpu.edu.cn/dc/c0/c5095a56512/page.htm).

### Statistical analysis

Continuous variables are described as the mean (standard deviation) and as the number (percent) for categorical variables. To compare the prognosis between patients who received RP and RT, multicovariate Cox regression models were used to calculate the hazard ratios (HRs) and 95% confidence intervals (CIs) for all-cause mortality. Age at diagnosis, TNM stage and grade were adjusted for in the SEER cohort. Age at diagnosis, TNM stage, grade, smoking status, education level, race, body mass index (BMI), aspirin dose, diabetes and family history were adjusted for in the PLCO cohort. The nonlinear relationship between diagnosed age, TNM stage and overall survival was detected by restricted cubic spline regression. All statistical analyses were performed using R (3.5.1).

## RESULTS

### Participant characteristics

**Figure 1:**
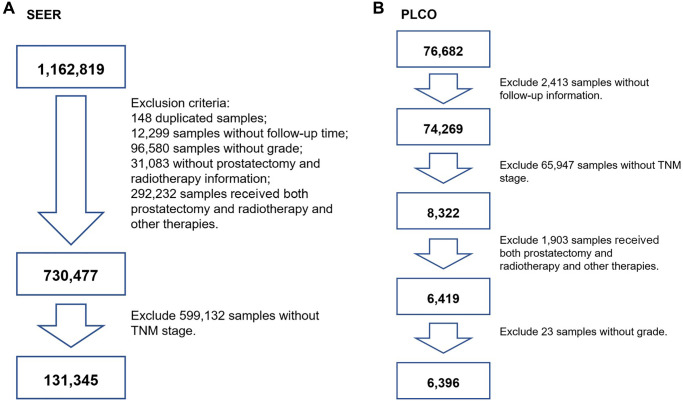
**The flowchart of screening participants using the SEER and PLCO cohort.** (**A**) for SEER screening, (**B**) for PLCO screening. TNM stage: tumor node metastasis stage.

For the SEER cohort (*n* = 1,162,819), 148 duplicated participants were initially excluded. Then, participants without follow-up time (*n* = 12,299), without grade (*n* = 96,580), without clinical TNM stage (*n* = 323,315), and without prostatectomy or radiotherapy (*n* = 31,083) information were excluded. Participants who received both prostatectomy and radiotherapy and those who received other therapies were excluded (*n* = 292,232). Next, patients with no information on TNM stage were excluded (*n* = 599,132). Eventually, 131,345 participants were included for further analysis. A flowchart of participant selection is shown in Figure 1A.

For the PLCO cohort (*n* = 76,682), we initially excluded participants who had a nonresponse form (*n* = 2,413). Participants without clinical TNM stage were also excluded (*n* = 65,947). Participants who received both prostatectomy and radiotherapy and those who received other therapies were excluded (*n* = 1,903). Then, patients with aberrant grade were excluded (*n* = 23). Finally, participants who underwent prostatectomy or received radiotherapy were used (*n* = 6,396). A flowchart is presented in Figure 1B.

There were 131,345 participants from the SEER cohort, including 74,663 (56.85%) men undergoing RP and 56,682 (43.16%) men who received RT. A total of 6,396 PCa patients were involved in the PLCO cohort, including 2,961 (46.27%) men treated with RP and 3,435 (53.73%) with RT. Information on the mean age at diagnosis, follow-up time and event-points that occurred in the RP and RT groups is presented in Table 1 for the SEER cohort and Table 2 for the PLCO cohort.

**Table 1:** Clinical and pathoanatomic characteristics of Surveillance, Epidemiology, and End results (SEER) (*n* = 131, 345).

**Variables**	**Prostatectomy (*n* = 74, 663)**	**Radiation (*n* = 56, 682)**
Age (years)	62.94 ± 8.54	67.53 ± 7.97
Survival time (months)	24.12 ± 13.84	24.83 ± 13.65
End-point events	2, 531 (3.39%)	2, 356 (4.16%)
TNM		
I	10, 092 (13.5%)	16, 279 (28.7%)
II	49, 470 (66.3%)	37, 042 (65.4%)
III	12, 507 (16.8%)	1, 410 (2.5%)
IV	2, 594 (3.5%)	1, 951 (3.4%)
Grade		
Well differentiated; Grade I	1, 179 (1.6%)	591 (1.0%)
Moderately differentiated; Grade II	25, 508 (34.2%)	20, 815 (36.7%)
Poorly differentiated; Grade III	47, 872 (64.1%)	35, 137 (62.0%)
Undifferentiated; anaplastic; Grade IV	104 (0.1%)	139 (0.2%)
T		
T1a	2, 682 (3.6%)	0 (0.0%)
T1b	1, 416 (1.9%)	0 (0.0%)
T1c	2, 228 (3.0%)	38, 171 (67.3%)
T1NOS	653 (0.9%)	0 (0.0%)
T2a	7, 797 (10.4%)	3, 917 (6.9%)
T2b	1, 420 (1.9%)	1, 934 (3.4%)
T2c	38, 918 (52.1%)	2, 085 (3.7%)
T2NOS	5, 275 (7.1%)	8, 418 (14.9%)
T3a	9, 890 (13.2%)	665 (1.2%)
T3b	3, 752 (5.0%)	605 (1.1%)
T3NOS	104 (0.1%)	446 (0.8%)
T4	476 (0.6%)	309 (0.5%)
TX	52 (0.1%)	132 (0.2%)
N		
N0	72, 789 (97.5%)	55, 652 (98.2%)
N1	1, 740 (2.3%)	840 (1.5%)
NX	134 (0.2%)	190 (0.3%)
M		
M0	73, 849 (98.9%)	55, 316 (97.6%)
M1a	54 (0.1%)	62 (0.1%)
M1b	563 (0.8%)	1, 039 (1.8%)
M1c	151 (0.2%)	232 (0.4%)
M1NOS	46 (0.1%)	33 (0.1%)

**Table 2:** Clinical and pathoanatomic characteristics the Prostate, Lung, Colorectal, and Ovarian (PLCO) (*n* = 6, 396).

**Variables**	**Prostatectomy (*n* = 2, 961)**	**Radiation (*n* = 3, 435)**
Age (years)	66.04 ± 4.59	70.50 ± 5.31
Survival time	144.13 ± 21.56	143.75± 22.31
End-point events	284	493
TNM		
I	4 (0.1%)	6 (0.2%)
II	2939 (99.3%)	3303 (96.2%)
III	17 (0.6%)	86 (2.5%)
IV	1 (0.0%)	40 (1.2%)
Grade		
Slight Anaplasia (G1)	77 (2.6%)	142 (4.1%)
Moderate Anaplasia (G2)	2447 (82.6%)	2774 (80.8%)
Marked Anaplasia (G3-4)	437 (14.8%)	519 (15.1%)
Gleason		
2–4	67 (2.3%)	110 (3.2%)
5–6	1416 (47.8%)	1834 (53.4%)
7–10	1456 (49.2%)	1471 (42.8%)
Not Available (99)	22 (0.7%)	20 (0.6%)
T		
T1 (3)	1 (0.0%)	0 (0.0%)
T1a (4)	21 (0.7%)	17 (0.5%)
T1b (5)	18 (0.6%)	22 (0.6%)
T1c (6)	1816 (61.3%)	2112 (61.5%)
T2 (7)	89 (3.0%)	46 (1.3%)
T2a (8)	619 (20.9%)	654 (19.0%)
T2b (9)	319 (10.8%)	443 (12.9%)
T2c (10)	61 (2.1%)	40 (1.2%)
T3 (11)	4 (0.1%)	23 (0.7%)
T3a (12)	8 (0.3%)	38 (1.1%)
T3b (13)	3 (0.1%)	19 (0.6%)
T3c (14)	2 (0.1%)	12 (0.3%)
T4 (15)	0 (0.0%)	7 (0.2%)
TX (1)	0 (0.0%)	2 (0.1%)
N		
N0 (2)	2261 (76.4%)	2899 (84.4%)
N1 (3)	0 (0.0%)	17 (0.5%)
N2 (4)	0 (0.0%)	0 (0.0%)
NX (1)	687 (23.2%)	512 (14.9%)
Not Available (99)	13 (0.4%)	7 (0.2%)
M		
M0 (2)	2532 (85.5%)	3066 (89.3%)
M1a (3)	0 (0.0%)	3 (0.1%)
M1b (4)	1 (0.0%)	18 (0.5%)
M1c (5)	0 (0.0%)	1 (0.0%)
MX (1)	421 (14.2%)	341 (9.9%)
Not Available (99)	7 (0.2%)	6 (0.2%)
PSA (ng/mL)		
0–4	496 (16.8%)	421 (12.3%)
4.1–10	1891 (63.9%)	2167 (63.1%)
10.1–20	353 (11.9%)	474 (13.8%)
20.1–50	65 (2.2%)	150 (4.4%)
50.1–100	9 (0.3%)	30 (0.9%)
100+	2 (0.1%)	16 (0.5%)
Not available	145 (4.9%)	177 (5.2%)
Smoke		
Never smoked	1031 (34.8%)	1124 (32.7%)
Current or former smoker	1861 (62.9%)	2218 (64.6%)
Not available	69 (2.3%)	93 (2.7%)
Education		
Less Than 8 Years	23 (0.8%)	37 (1.1%)
8-11 Years	150 (5.1%)	226 (6.6%)
12 Years or Completed High School	530 (17.9%)	583 (17.0%)
Post High School Training Other Than College	343 (11.6%)	375 (10.9%)
Some College	556 (18.8%)	704 (20.5%)
College Graduate	593 (20.0%)	631 (18.4%)
Postgraduate	689 (23.3%)	783 (22.8%)
Not available	77 (2.6%)	96 (2.8%)
Race		
White, Non-Hispanic	2648 (89.4%)	2893 (84.2%)
Black, Non-Hispanic	138 (4.7%)	234 (6.8%)
Hispanic	45 (1.5%)	64 (1.9%)
Asian	42 (1.4%)	123 (3.6%)
Pacific Islander	10 (0.3%)	23 (0.7%)
American Indian	7 (0.2%)	5 (0.1%)
Missing	71 (2.4%)	93 (2.7%)
BMI	27.22 ± 3.68	27.41 ± 4.02
Aspirin		
None	1392 (47.0%)	1496 (43.6%)
1/Day	623 (21.0%)	879 (25.6%)
2+/Day"	136 (4.6%)	147 (4.3%)
1/Week	38 (1.3%)	38 (1.1%)
2/Week	99 (3.3%)	122 (3.6%)
3-4/Week	297 (10.0%)	306 (8.9%)
<2/Month	195 (6.6%)	220 (6.4%)
2-3/Month	107 (3.6%)	123 (3.6%)
Not available	74 (2.5%)	104 (3.0%)
Diabetes		
No	2737 (92.4%)	3073 (89.5%)
YES	137 (4.6%)	249 (7.2%)
Not available	87 (2.9%)	113 (3.3%)
Family history of Prostate Cancer		
No	2481 (83.8%)	2927 (85.2%)
Yes, Immediate Family Member	349 (11.8%)	346 (10.1%)
Possibly - Relative Or Cancer Type Not Clear	44 (1.5%)	51 (1.5%)
Not available	87 (2.9%)	111 (3.2%)
Comorbidity		
0	1209 (40.8%)	1103 (32.1%)
1	989 (33.4%)	1163 (33.9%)
2	516 (17.4%)	698 (20.3%)
≥3	247 (8.3%)	471 (13.7%)

### Survival analysis

In the SEER cohort, patients treated with RT showed a worse outcome than those who underwent RP (HR: 1.09, 95% CI: 1.02–1.16, *p* = 0.0069) (Table 3). This significant association was validated in the PLCO cohort (HR: 1.46, 95% CI: 1.24–1.72, *p* = < 0.0001) (Table 3). Patients who received both prostatectomy and radiotherapy versus only prostatectomy or radiotherapy in the PLCO cohort showed no significant associations (Supplementary Table 1).

**Table 3:** Hazard ratios (HRs) with 95% confidence intervals (95% CIs) of prostate cancer (PCa) deaths for men who received radiotherapy (RT) versus those who underwent radical prostatectomy (RP) in SEER and PLCO.

**Dataset**	**No. of patients (deaths/total)**	**HR**	**95%CI**	***p***	**HR**	**95%CI**	***p***
SEER	4887/131,345	1.19	1.13–1.26	< 0.0001^a^	1.09	1.02–1.16	0.0069^b^
PLCO	777/6,396	1.50	1.30–1.73	< 0.0001^a^	1.46	1.24–1.72	< 0.0001^c^

^a^unadjusted.

^b^adjusted age at diagnosis (5-year groups), tumor node metastasis (TNM) stage (continuous) and grade (continuous).

^c^adjusted age at diagnosis (5-year groups), TNM stage (continuous), grade (continuous), smoke (categorical), education levels (continuous), race (categorical), body mass index (continuous), aspirin does (continuous), diabetes (categorical) and family history (categorical).

Abbreviations: SEER, Surveillance, Epidemiology, and End Results; PLCO, the Prostate, Lung, Colorectal, and Ovarian.

In the next step, stratified analysis was performed according to clinical TNM stage to compare the treatment outcomes of RP and RT for PCa patients in the SEER cohort (Table 4). Of note, PCa patients at TNM stages I, IIA and IIB showed better survival with RT treatment than RP patients (*p* = < 0.0001 for stage I, *p* = < 0.0001 for stage IIA and *p* = 0.0048 for stage IIB). In contrast, those at TNM stages III and IV showed worse prognosis with RT treatment compared with RP (*p* = 0.026 for stage III and *p* = < 0.0001 for stage IV).

**Table 4:** Hazard ratios (HRs) with 95% confidence intervals (95% CIs) of prostate cancer (PCa) deaths for men who received radiotherapy (RT) versus those who underwent radical prostatectomy (RP) in SEER stratified by TNM stage.

**TNM stage**	**No. of patients (deaths/total)**	**HR^a^**	**95%CI^a^**	***p*^a^**	**HR^b^**	**95%CI^b^**	***p*^b^**
I	984/26,371	0.31	0.27–0.35	< 0.0001	0.35	0.30–0.40	*<* 0.0001
IIA	1056/32,631	0.82	0.72–0.93	0.0025	0.67	0.58–0.76	*<* 0.0001
IIB	1547/53,881	2.15	1.94–2.38	< 0.0001	0.85	0.76–0.95	0.0048
III	299/13,917	2.86	2.20–3.71	< 0.0001	1.38	1.04–1.84	0.026
IV	1001/4,545	1.83	1.61–2.07	< 0.0001	1.78	1.59–2.05	*<* 0.0001

^a^unadjusted.

^b^adjusted age at diagnosis (5-year groups) and grade (continuous).

Abbreviation: SEER, Surveillance, Epidemiology, and End Results.

Then, stratified analysis was performed according to age at diagnosis in the SEER cohort (Table 5). PCa patients treated with RT showed a better prognosis than those who underwent RP in the 75–79, 80–84 and 85+ year age groups (*p* < 0.05) but a worse prognosis in the 45–49, 50–54, 55–59, 60–64, and 65–69 year age groups (*p* < 0.05). There were no significant differences for patients in the age range of 70–74 years in the comparison between RP and RT.

**Table 5:** Hazard ratios (HRs) with 95% confidence intervals (95% CIs) of prostate cancer (PCa) deaths for men who received radiotherapy (RT) versus those who underwent radical prostatectomy (RP) in SEER stratified by age groups.

**Age (years)**	**No. of patients (deaths/total)**	**HR^a^**	**95%CI^a^**	***p*^a^**	**HR^b^**	**95%CI^b^**	***p*^b^**
45–49	46/3,461	3.80	2.13–6.79	< 0.0001	6.40	3.50–11.68	< 0.0001
50–54	179/10,892	3.09	2.30–4.14	< 0.0001	5.32	3.95–7.16	< 0.0001
55–59	347/19,949	2.43	1.97–3.00	< 0.0001	4.04	3.22–5.06	< 0.0001
60–64	611/27,645	1.92	1.64–2.25	< 0.0001	3.36	2.84–3.98	< 0.0001
65–69	861/30,593	1.36	1.19–1.56	< 0.0001	2.15	1.86–2.49	< 0.0001
70–74	864/20,527	0.77	0.68–0.88	0.0002	1.09	0.94–1.27	0.21
75–79	808/11,239	0.42	0.37–0.49	< 0.0001	0.52	0.45–0.61	< 0.0001
80–84	658/4,414	0.32	0.28–0.38	< 0.0001	0.34	0.29–0.40	< 0.0001
85+	504/1,767	0.44	0.36–0.55	< 0.0001	0.39	0.32–0.49	< 0.0001

^a^unadjusted.

^b^adjusted tumor node metastasis (TNM) stage (continuous) and grade (continuous).

Abbreviation: SEER, Surveillance, Epidemiology, and End Results.

**Figure 2:**
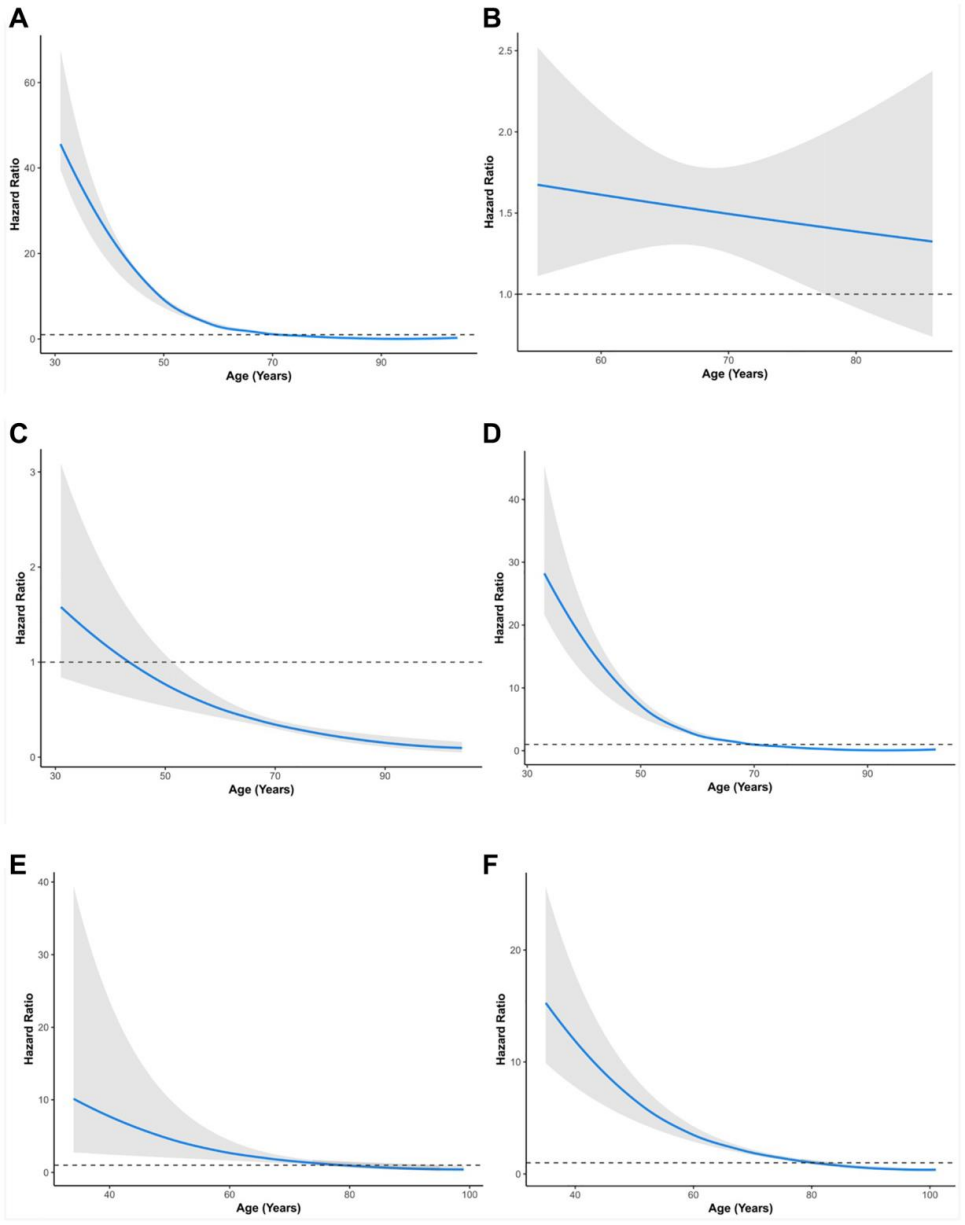
**Relationship between age and hazard ratio of RT versus RP for patients with prostate cancer.** (**A**) All groups in SEER. (**B**) All groups in PLCO. (**C**) Stage I group in SEER. (**D**) Stage II group in SEER. (**E**) Stage III group in SEER. (**F**) Stage IV group in SEER. SEER, Surveillance, Epidemiology, and End Results; PLCO, the Prostate, Lung, Colorectal, and Ovarian. The solid lines are multivariable adjusted hazard ratios. The shaded areas are the 95% confidence intervals. The dotted lines are coordinate 1 on the vertical axis.

Subsequently, TNM stage and age were simultaneously considered for stratified analysis. The HR of RT versus RP in overall survival decreased with age (Figure 2A, Table 6). This negative correlation was also found in stages I, IIA, and IIB in the SEER cohort (Figure 2C, 2D, 2E, 2F, Table 6).

**Table 6:** Hazard ratios (HRs) with 95% confidence intervals (95% CIs) of prostate cancer (PCa) death for men who received radiotherapy (RT) versus those who underwent radical prostatectomy (RP) in SEER stratified by age according to TNM stage.

**TNM stage**	**Age (years)**	**No. of patients (deaths/total)**	**HR^a^**	**95% CI^a^**	***p*^a^**	**HR^b^**	**95% CI^b^**	***p*^b^**
Stage I	45–49	11/669	0.97	0.3–3.19	0.96	0.99	0.29–3.30	0.99
	50–54	32/1, 994	0.85	0.42–1.7	0.64	0.87	0.43–1.74	0.69
	55–59	76/3, 879	0.60	0.38–0.94	0.026	0.59	0.38–0.93	0.022
	60–64	117/5, 520	0.48	0.33–0.69	< 0.0001	0.47	0.33–0.68	< 0.0001
	65–69	163/6, 239	0.40	0.29–0.54	< 0.0001	0.40	0.29–0.54	< 0.0001
	70–74	172/4, 302	0.25	0.18–0.34	< 0.0001	0.25	0.18–0.34	< 0.0001
	75–79	173/2, 321	0.29	0.21–0.39	< 0.0001	0.29	0.21–0.39	< 0.0001
	80–84	145/879	0.37	0.24–0.57	< 0.0001	0.37	0.24–0.57	< 0.0001
	85+	93/393	0.22	0.05–0.89	0.034	0.20	0.05–0.83	0.026
Stage IIA	45–49	6/456	2.56	0.47–14.01	0.28	2.54	0.46–14.01	0.28
	50–54	23/1, 697	1.23	0.54–2.81	0.62	1.25	0.55–2.86	0.59
	55–59	62/3, 587	2.96	1.54–5.69	0.0011	2.93	1.53–5.63	0.0012
	60–64	130/6, 089	2.03	1.30–3.16	0.0017	2.04	1.31–3.18	0.0016
	65–69	207/7, 902	0.98	0.71–1.34	0.89	0.98	0.71–1.34	0.89
	70–74	205/6, 733	0.58	0.43–0.80	0.0009	0.58	0.43–0.80	0.0009
	75–79	203/4, 245	0.58	0.41–0.81	0.0016	0.58	0.41–0.82	0.0021
	80–84	135/1, 430	0.29	0.21–0.41	< 0.0001	0.29	0.21–0.41	< 0.0001
	85+	83/388	0.41	0.25–0.67	0.0007	0.41	0.25–0.67	0.0004
stage IIB	45–49	13/1, 910	2.28	0.30–17.54	0.43	2.28	0.28–18.39	0.44
	50–54	50/5, 645	2.54	1.14–5.64	0.022	2.45	1.09–5.55	0.031
	55–59	107/9, 536	2.54	1.59–4.06	< 0.0001	2.34	1.45–3.80	0.0005
	60–64	173/11, 768	3.35	2.46–4.56	< 0.0001	3.52	2.55–4.85	< 0.0001
	65–69	260/11, 799	2.20	1.72–2.82	< 0.0001	2.17	1.69–2.81	< 0.0001
	70–74	283/6, 910	1.37	1.09–1.73	0.0078	1.39	1.09–1.76	0.0074
	75–79	266/3, 582	0.49	0.38–0.62	< 0.0001	0.52	0.40–0.67	< 0.0001
	80–84	210/1, 594	0.23	0.18–0.30	< 0.0001	0.24	0.18–0.31	< 0.0001
	85+	182/645	0.25	0.17–0.36	< 0.0001	0.25	0.17–0.36	< 0.0001
Stage III	45–49	2/333	–	–	–	–	–	1.0
	50–54	8/1, 220	3.04	0.37–24.8	0.30	2.99	0.36–24.58	0.31
	55–59	24/2, 351	5.02	1.99–12.66	0.0006	4.79	1.89–12.12	0.0009
	60–64	51/3, 416	1.57	0.62–3.96	0.34	1.54	0.61–3.87	0.36
	65–69	67/3, 691	2.17	1.14–4.14	0.019	2.21	1.16–4.23	0.016
	70–74	60/1, 933	2.37	1.38–4.05	0.0017	2.36	1.38–4.04	0.0017
	75–79	35/631	0.70	0.34–1.47	0.35	0.70	0.33–1.45	0.34
	80–84	31/203	0.47	0.23–0.96	0.038	0.48	0.23–0.97	0.041
	85+	21/78	0.50	0.2–1.27	0.15	0.50	0.20–1.27	0.15
Stage IV	45–49	14/93	5.91	1.65–21.22	0.0064	5.85	1.63–21.04	0.0068
	50–54	66/336	3.84	2.23–6.6	< 0.0001	3.84	2.24–6.61	< 0.0001
	55–59	78/596	5.18	3.12–8.62	< 0.0001	5.19	3.11–8.66	< 0.0001
	60–64	140/852	2.83	1.99–4.02	< 0.0001	2.84	2.00–4.03	< 0.0001
	65–69	164/962	2.83	2.05–3.91	< 0.0001	2.83	2.05–3.92	< 0.0001
	70–74	144/649	1.66	1.18–2.32	0.0032	1.66	1.19–2.33	0.003
	75–79	131/460	0.88	0.63–1.25	0.48	0.88	0.62–1.24	0.47
	80–84	137/308	1.15	0.82–1.61	0.42	1.21	0.86–1.71	0.28
	85+	125/263	0.80	0.55–1.17	0.25	0.80	0.55–1.16	0.24

^a^unadjusted.

^b^adjusted grade (continuous).

Abbreviation: SEER, Surveillance, Epidemiology, and End Results.

Additionally, this finding was validated in the PLCO cohort (Figure 2B). Specifically, in TNM stage I, patients aged 55–85+ years (HR: 0.20–0.59, *p* < 0.05) showed better survival with RT than RP patients (Table 6). In TNM stage IIA, patients aged 70–85+ years (HR: 0.41–0.58, *p* < 0.05) showed better survival with RT than RP. In contrast, patients aged 55–64 years (HR: 2.04–2.93, *p* < 0.05) showed worse survival with RT than with RP (Table 6). In TNM stage IIB patients, those aged 75–85+ years (HR: 0.25–0.52, *p* < 0.05) showed better survival with RT than RP patients. In contrast, patients aged 50–74 years (HR: 1.39–2.45, *p* < 0.05) showed worse survival with RT than with RP (Table 6). In TNM stage III, patients aged 80–84 years (HR: 0.48, 95% CI: 0.23–0.97, *p* = 0.041) showed better survival with RT than RP patients. In contrast, patients aged 55–74 years (HR: 2.36–4.79, *p* < 0.05) showed worse survival with RT than with RP (Table 6). In TNM stage IV, patients aged 45–74 years (HR: 1.66–5.85, *p* < 0.05) showed worse survival with RT than with RP. PCa patients in other age groups within stages showed no significant differences between RP and RT (Table 6).

## DISCUSSION

In this study, we compared the prognosis of PCa patients with RT and RP in two databases involving more than 130,000 subjects. We investigated the different prognoses of discrepant treatments using the SEER database. Furthermore, the results were confirmed in the PLCO database. Compared to RP, as age increases, the prognosis of RT improves, while as the malignancy of PCa increases, the prognosis of RT worsens. For each patient, our study suggested better treatment according to age and TNM stage information. In the last decade, many studies have been conducted to compare the prognosis after RT and RP. Petrelli et al. summarized 17 studies and conducted a meta-analysis. The results found lower overall mortality and cancer-related mortality after RP than after RT [14]. Moreover, another meta-analysis including 19 studies also indicated that overall and prostate cancer-specific mortality for patients treated with RT is higher than that for patients treated with RP [5]. However, a large-sample observational study suggested that the difference in prognosis after RT and RP was quite small [7]. In line with this result, Hamdy and his colleagues also reported that prostate cancer-specific mortality was low irrespective of the treatment assigned after a median of 10 years [6]. Similar results were observed in patients older than 65 [15]. Two other studies supported no difference between the choice of RT and RP [16, 17].

Through stratified analysis rather than adjustment, we found that as the age and cancer process are altered, the best treatment for patients changes. A large-sample cohort study showed similar results after a 13-year follow-up [18]. However, because their cohorts consisted of patients from 65 to 80 years old, divided into three categories, no significant trend of HRs with age was shown. Moreover, the conclusion was drawn by three additional studies that younger men and those with intermediate- or high-risk localized PCa might have a greater benefit from RP [19–21]. These elucidations are consistent with our results. Our result is different but does not conflict with previous studies. Age and TNM stage were adjusted for in previous studies, but few stratified analyses were performed. Thus, the trend by age and TNM stage for HRs for RT versus RP is likely to be covered. Meanwhile, due to differences in the age composition and progression of PCa, different cohorts may lead to different results. Our results may demonstrate why previous studies drew different conclusions.

As a retrospective study, straightforward comparisons of survival between different treatments would be biased by confounders. In this work, we have taken several measures to reduce the bias. First, we established strict inclusion and exclusion criteria for participants. Second, stratified analyses were performed rather than adjustment. To eliminate potential confounders, available baseline characteristics such as grade and BMI were included in the model. Third, two independent PCa cohorts were employed to confirm the results. The significant interaction between TNM stage/age and treatment in survival in two cohorts indicated that there were associations between them. The results changed little when other factors were included in the models. Thus, potential confounders may result in little bias. To further validate the findings, rigorous and long-term randomized controlled trials are needed before clinical use.

The limitations of our study include the following three aspects. First, our results were obtained from public databases rather than independent cohorts. Second, the cohorts from the two databases were heterogeneous to some extent, and the sample size of PLCO was relatively small. This made the results from the two databases not completely consistent. Third, because of restricted available information on subjects in the SEER database, we only adjusted for age, grade and TNM stage.

In summary, this study highlights a difference in the prognosis of PCa patients between RT and RP based on stratified analysis by TNM stage and age. Thus, the choice between these two treatments should be guided by TNM stage and age. These findings may facilitate counseling regarding the prognostic effect of RT and RP.

## Supplementary Materials

Supplementary Table 1

## References

[1] Siegel RL, Miller KD, Jemal A. Cancer statistics, 2018. CA Cancer J Clin. 2018; 68:7–30. 10.3322/caac.21442 29313949

[2] Liu S, Yang L, Yuan Y, Li H, Tian J, Lu S, Wang N, Ji J. Cancer incidence in Beijing, 2014. Chin J Cancer Res. 2018; 30:13–20. 10.21147/j.issn.1000-9604.2018.01.02 29545715 PMC5842227

[3] Widmark A, Klepp O, Solberg A, Damber JE, Angelsen A, Fransson P, Lund JA, Tasdemir I, Hoyer M, Wiklund F, Fosså SD, and Scandinavian Prostate Cancer Group Study 7, and Swedish Association for Urological Oncology 3. Endocrine treatment, with or without radiotherapy, in locally advanced prostate cancer (SPCG-7/SFUO-3): an open randomised phase III trial. Lancet. 2009; 373:301–08. 10.1016/S0140-6736(08)61815-2 19091394

[4] Holmberg L, Bill-Axelson A, Helgesen F, Salo JO, Folmerz P, Häggman M, Andersson SO, Spångberg A, Busch C, Nordling S, Palmgren J, Adami HO, Johansson JE, Norlén BJ, and Scandinavian Prostatic Cancer Group Study Number 4. A randomized trial comparing radical prostatectomy with watchful waiting in early prostate cancer. N Engl J Med. 2002; 347:781–89. 10.1056/NEJMoa012794 12226148

[5] Wallis CJD, Saskin R, Choo R, Herschorn S, Kodama RT, Satkunasivam R, Shah PS, Danjoux C, Nam RK. Surgery Versus Radiotherapy for Clinically-localized Prostate Cancer: A Systematic Review and Meta-analysis. Eur Urol. 2016; 70:21–30. 10.1016/j.eururo.2015.11.010 26700655

[6] Hamdy FC, Donovan JL, Lane JA, Mason M, Metcalfe C, Holding P, Davis M, Peters TJ, Turner EL, Martin RM, Oxley J, Robinson M, Staffurth J, et al, and ProtecT Study Group. 10-Year Outcomes after Monitoring, Surgery, or Radiotherapy for Localized Prostate Cancer. N Engl J Med. 2016; 375:1415–24. 10.1056/NEJMoa1606220 27626136

[7] Robinson D, Garmo H, Lissbrant IF, Widmark A, Pettersson A, Gunnlaugsson A, Adolfsson J, Bratt O, Nilsson P, Stattin P. Prostate Cancer Death After Radiotherapy or Radical Prostatectomy: A Nationwide Population-based Observational Study. Eur Urol. 2018; 73:502–11. 10.1016/j.eururo.2017.11.039 29254629

[8] Hoffman RM, Koyama T, Fan KH, Albertsen PC, Barry MJ, Goodman M, Hamilton AS, Potosky AL, Stanford JL, Stroup AM, Penson DF. Mortality after radical prostatectomy or external beam radiotherapy for localized prostate cancer. J Natl Cancer Inst. 2013; 105:711–18. 10.1093/jnci/djt059 23615689 PMC3653822

[9] Lei JH, Liu LR, Wei Q, Yan SB, Song TR, Lin FS, Yang L, Cao DH, Yuan HC, Xue WB, Lv X, Cai YC, Zeng H, Han P. Systematic review and meta-analysis of the survival outcomes of first-line treatment options in high-risk prostate cancer. Sci Rep. 2015; 5:7713. 10.1038/srep07713 25578739 PMC5378991

[10] Abdollah F, Sun M, Thuret R, Jeldres C, Tian Z, Briganti A, Shariat SF, Perrotte P, Rigatti P, Montorsi F, Karakiewicz PI. A competing-risks analysis of survival after alternative treatment modalities for prostate cancer patients: 1988-2006. Eur Urol. 2011; 59:88–95. 10.1016/j.eururo.2010.10.003 20965646

[11] Dong X, Ma G, Chen F. Age at diagnosis and prognosis among prostate cancer patients treated with radiotherapy: evidenced from three independent cohort studies. Ann Oncol. 2018; 29:2019–20. 10.1093/annonc/mdy234 29992296

[12] Duggan MA, Anderson WF, Altekruse S, Penberthy L, Sherman ME. The Surveillance, Epidemiology, and End Results (SEER) Program and Pathology: Toward Strengthening the Critical Relationship. Am J Surg Pathol. 2016; 40:e94–102. 10.1097/PAS.0000000000000749 27740970 PMC5106320

[13] Prorok PC, Andriole GL, Bresalier RS, Buys SS, Chia D, Crawford ED, Fogel R, Gelmann EP, Gilbert F, Hasson MA, Hayes RB, Johnson CC, Mandel JS, et al, and Prostate, Lung, Colorectal and Ovarian Cancer Screening Trial Project Team. Design of the Prostate, Lung, Colorectal and Ovarian (PLCO) Cancer Screening Trial. Control Clin Trials. 2000 (Suppl 6); 21:273S–309S. 10.1016/s0197-2456(00)00098-2 11189684

[14] Petrelli F, Vavassori I, Coinu A, Borgonovo K, Sarti E, Barni S. Radical prostatectomy or radiotherapy in high-risk prostate cancer: a systematic review and metaanalysis. Clin Genitourin Cancer. 2014; 12:215–24. 10.1016/j.clgc.2014.01.010 24589471

[15] Gu X, Gao X, Cui M, Xie M, Ma M, Qin S, Li X, Qi X, Bai Y, Wang D. Survival outcomes of radical prostatectomy and external beam radiotherapy in clinically localized high-risk prostate cancer: a population-based, propensity score matched study. Cancer Manag Res. 2018; 10:1061–67. 10.2147/CMAR.S157442 29773955 PMC5947109

[16] Tamada S, Ninomiya N, Kitamoto K, Kato M, Yamasaki T, Iguchi T, Ohmachi T, Nakatani T. Comparative effectiveness of radical prostatectomy and curative radiotherapy in localized prostate cancer: long-term follow-up. J Radiat Res. 2017; 58:552–58. 10.1093/jrr/rrw119 28013228 PMC5570081

[17] Akakura K, Suzuki H, Ichikawa T, Fujimoto H, Maeda O, Usami M, Hirano D, Takimoto Y, Kamoto T, Ogawa O, Sumiyoshi Y, Shimazaki J, Kakizoe T, and Japanese Study Group for Locally Advanced Prostate Cancer. A randomized trial comparing radical prostatectomy plus endocrine therapy versus external beam radiotherapy plus endocrine therapy for locally advanced prostate cancer: results at median follow-up of 102 months. Jpn J Clin Oncol. 2006; 36:789–93. 10.1093/jjco/hyl115 17082219

[18] Abdollah F, Schmitges J, Sun M, Jeldres C, Tian Z, Briganti A, Shariat SF, Perrotte P, Montorsi F, Karakiewicz PI. Comparison of mortality outcomes after radical prostatectomy versus radiotherapy in patients with localized prostate cancer: a population-based analysis. Int J Urol. 2012; 19:836–44. 10.1111/j.1442-2042.2012.03052.x 22574746

[19] Sooriakumaran P, Nyberg T, Akre O, Steineck G, Wiklund P. Authors' reply to Roach. BMJ. 2014; 348:g2271. 10.1136/bmj.g2271 24668778

[20] Bill-Axelson A, Holmberg L, Ruutu M, Garmo H, Stark JR, Busch C, Nordling S, Häggman M, Andersson SO, Bratell S, Spångberg A, Palmgren J, Steineck G, et al, and SPCG-4 Investigators. Radical prostatectomy versus watchful waiting in early prostate cancer. N Engl J Med. 2011; 364:1708–17. 10.1056/NEJMoa1011967 21542742

[21] Wilt TJ, Brawer MK, Jones KM, Barry MJ, Aronson WJ, Fox S, Gingrich JR, Wei JT, Gilhooly P, Grob BM, Nsouli I, Iyer P, Cartagena R, et al, and Prostate Cancer Intervention versus Observation Trial (PIVOT) Study Group. Radical prostatectomy versus observation for localized prostate cancer. N Engl J Med. 2012; 367:203–13. 10.1056/NEJMoa1113162 22808955 PMC3429335

