# Supplementary material for: Prognostic comparison between radical prostatectomy and radiotherapy in prostate cancer patients at different stages and ages
Source: Aging (Albany NY). 2021 Jun 29;13(12):16773–85. doi: 10.18632/aging.203198 (PMC8266375; doi:10.18632/aging.203198)
Supplement: Supplementary Table 1 [file aging-13-203198-s001.pdf]

SUPPLEMENTARY TABLE

Supplementary Table 1. Hazard ratios (HRs) with 95% confidence intervals (95% CIs) of prostate cancer (PCa) deaths for men received both prostatectomy and radiotherapy versus only prostatectomy or radiotherapy in PLCO.

PLCO	No. of patients (deaths/total)	HR[a]	95% CI[a]	p^a	HR[b]	95% CI[b]	p^b
Both prostatectomy and radiotherapy Verse prostatectomy	301/3, 105	1.23	0.75–2.00	0.42	1.13	0.67–1.90	0.64
Both prostatectomy and radiotherapy Verse radiotherapy	510/3, 579	0.82	0.50–1.33	0.41	0.83	0.50–1.38	0.47

[a]unadjusted.
[b]adjusted age at diagnosis (5-year groups), TNM stage (continuous), grade (continuous), smoke (categorical), education levels (continuous), race (categorical), body mass index (continuous), aspirin does (continuous), diabetes (categorical) and family history (categorical).
Abbreviation: PLCO, the Prostate, Lung, Colorectal, and Ovarian.
